# The quality of care and long-term mortality of patients with ST-elevation myocardial infarction and cardiac devices: a nationwide cohort study

**DOI:** 10.1093/ehjopen/oeaf139

**Published:** 2025-10-23

**Authors:** Nicholas Weight, Balamrit Singh Sokhal, Muhammad Rashid, Mohamed Dafaalla, Christian D Mallen, Mamas A Mamas

**Affiliations:** Keele Cardiovascular Research Group, Keele University, Keele, Staffordshire, ST5 5BH, UK; Keele Cardiovascular Research Group, Keele University, Keele, Staffordshire, ST5 5BH, UK; School of Medicine, Keele University, Keele, Staffordshire, ST5 5BG, UK; Royal Stoke University Hospital, University Hospitals North Midlands, Stoke-On-Trent, Staffordshire, ST4 6QG, UK; Keele Cardiovascular Research Group, Keele University, Keele, Staffordshire, ST5 5BH, UK; Department of Cardiovascular Sciences, University of Leicester, Leicester, LE1 7RH, UK; National Institute for Health Research (NIHR) Leicester Cardiovascular Biomedical Research Unit, Glenfield Hospital, Leicester, LE3 9QP, UK; Keele Cardiovascular Research Group, Keele University, Keele, Staffordshire, ST5 5BH, UK; School of Medicine, Keele University, Keele, Staffordshire, ST5 5BG, UK; Keele Cardiovascular Research Group, Keele University, Keele, Staffordshire, ST5 5BH, UK; National Institute for Health and Care Research (NIHR) Birmingham Biomedical Research Centre, Birmingham, B15 2TH, UK

**Keywords:** ST-elevation myocardial infarction, Pacemaker, Quality of care, Long-term mortality

## Abstract

**Introduction:**

There is a growing population with cardiac devices (pacemakers, implantable cardioverter defibrillators and cardiac resynchronization therapy), but whether this influences quality of care and long-term mortality after ST-elevation myocardial infarction (STEMI) is unknown.

**Methods and results:**

Patients in England and Wales between January 2005 and March 2019 with a diagnosis of STEMI were included from the Myocardial Ischaemia National Audit Project, Hospital Episode Statistics and mortality linkage to July 2021. Primary outcomes were all-cause mortality over the study period, secondary outcomes were odds of undergoing reperfusion within guideline mandated timeframes. Multivariate cox-models compared all-cause mortality over specified time-periods and logistic regression models illustrated odds of undergoing reperfusion. 322 890 patients with STEMI were included, 2118 (0.7%) had a cardiac device at STEMI admission. Patients with cardiac devices were older (78 years old vs. 66 years old) and more often female (32% vs. 29%) (*P* < 0.001). After multivariate adjustment, patients with cardiac devices were less likely to have a ‘door-to-balloon time’ of under 60 min (aOR 0.61 95% CI 0.54–0.70) (*P* < 0.001). Patients with cardiac devices had an increased risk of all-cause mortality at 5-years (aHR 1.12 95% CI 1.05–1.20) (*P* < 0.001). Excluding patients dying within 30 days of admission, patients with cardiac devices still had a higher risk of death at 5-years (aHR 1.23 95% CI 1.13–1.33) (all *P* < 0.001).

**Conclusion:**

Patients with cardiac devices were less likely to undergo revascularization for STEMI within guideline mandated timeframes. They remain at elevated risk of all-cause mortality up to 5-years compared with STEMI patients without cardiac devices.

## Graphical Abstract

**Graphical Abstract:**
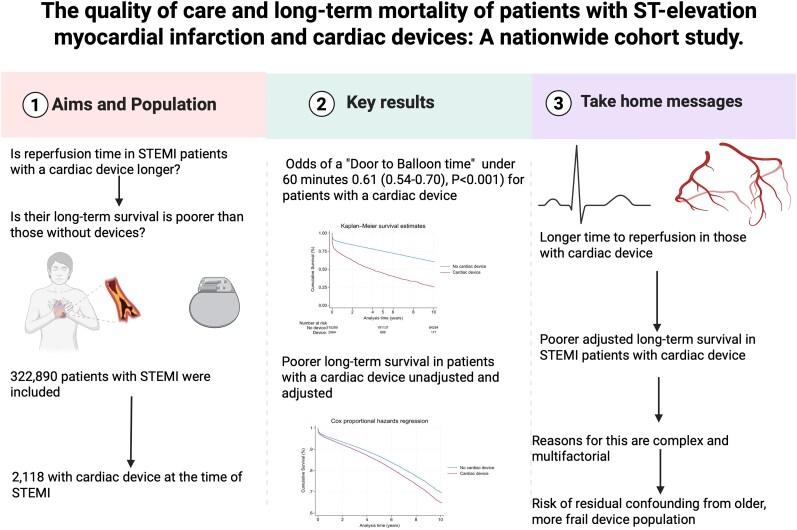

## Introduction

Cardiovascular disease (CVD) is the leading cause of mortality and morbidity worldwide, with acute coronary syndrome (ACS) representing the primary driver of cardiovascular death.^1^ In patients with established ST-elevation myocardial infarction (STEMI), contemporary guidelines recommend prompt reperfusion with primary percutaneous coronary intervention (PPCI) within 120 min from first contact with medical services,^2^ given that delays to reperfusion have been clearly demonstrated to lead to poorer long-term survival, poorer residual left ventricular function and more mechanical complications.^3,4^

Implanted cardiac devices, comprising permanent pacemakers, cardiac resynchronization therapy defibrillators (CRT-D), cardiac resynchronization therapy (CRT-P) pacemakers and implantable cardioverter-defibrillator (ICD), are increasingly prevalent given the increase of incident CVD in an ageing population.^5^ In a Western Australian population, over 1 in 50 people aged 75 years or older have a permanent pacemaker (PPM) inserted.^5^ The presence of a PPM, specifically a paced rhythm, can make diagnosis of STEMI challenging, given right ventricular (RV) pacing induces a left bundle branch block pattern (LBBB) on ECG.^6^ Criteria exist to diagnose STEMI in patients with LBBB, such as the Smith-Modified Sgarbossa criteria, but awareness of this is not yet universal,^7^ and therefore guideline mandated PPCI may be delayed in this patient cohort.^2^ Few studies have characterized the time to reperfusion and longer-term outcomes of patients with cardiac devices admitted with STEMI, and the small number that do commonly have had limited population sizes.^6,8^

Using a large national heart attack registry, this study aims to examine long-term all-cause mortality in STEMI patients with implanted cardiac devices, and to evaluate in-hospital quality of care—specifically the likelihood of receiving PPCI within guideline-recommended timeframe.

## Methods

### Study design

This study was retrospective cohort study of prospectively collected data from the Myocardial Ischaemia National Audit Project (MINAP) linked to the Office for National Statistics (ONS) and Hospital Episode Statistics (HES). MINAP is a prospective national registry of patients with ACS admitted to 230 hospitals in the United Kingdom (UK). MINAP includes data on patient demographics, clinical characteristics, comorbidities, pharmacotherapy, management and in-hospital outcomes, on ∼90 000 patients admitted with ACS per year. The ONS is the independent provider of mortality statistics in the UK, collecting data on all deaths registered in England and Wales using *International Classification of Diseases, Tenth Revision (ICD-10)* codes, and cause of death from the Medical Certificate of Cause of Death. The ONS allows for long-term mortality data to be investigated in conjunction with MINAPs short term outcomes. Mortality follow-up was available for all included patients up to July 2021.

### Study population

Using the ICD-10 code (Z95.0), the ‘presence of an electronic cardiac device’, which comprises; cardiac pacemaker, cardiac resynchronization therapy defibrillator (CRT-D), cardiac resynchronization therapy (CRT-P) pacemaker and ICD, all index admissions between January 2005 and March 2019 with a primary diagnosis of STEMI were identified and stratified according to the presence of an implanted cardiac device. Z95.0 does not include any temporary transvenous pacemakers inserted. Implanted cardiac device comprised PPM, implantable cardioverter defibrillator (ICD) and cardiac resynchronization therapy (CRT). Diagnosis of atrial fibrillation (AF) or atrial flutter was extract by the ICD-10 code I48. The discharge diagnosis of STEMI was determined by local clinicians according to presenting history, clinical examination, and the results of in-patient investigations in keeping with the consensus document of the Joint European Society of Cardiology (ESC) and American College of Cardiology.^9^ Patients were excluded if there was missing data for variables such as in-hospital mortality, major adverse cardiovascular events, cause of death or missing unique identifier, which is the patient National Health Service (NHS) number. The first admission with STEMI over our study period for each patient is included, with duplicate records or readmissions over the study period identified and removed using date of admission and NHS number. Patients with a procedure code in their HES record that suggests a cardiac device was inserted during the admission with STEMI were not included in the analysis.

### Outcomes

The primary outcomes were all-cause mortality, over the entire study period, which ended in July 2021, which we referred to as ‘overall mortality’ and additionally displayed at 30 days, 1-year and 5-years. Secondary outcomes of interest were quality of care measures, including the Opportunity Based Quality Indicators (OBQI), which consists of prescription of aspirin, P2Y12 inhibitors, beta-blockers, statins, ACE inhibitors/ARBs and whether referral to cardiac rehabilitation was made while an inpatient. Additionally, guideline mandated time to definitive reperfusion therapy, specifically ‘Door to Balloon time’, which comprises time from arrival at hospital to first coronary balloon inflation, was assessed at 60 min and 90 min and ‘Call to Balloon time’, which comprises time from patient initiated ‘Call for help’, which is time of call to ambulances services, to time of coronary balloon inflation, was assessed at 120 min.

### Statistical analysis

Continuous variables such as age at admission, and body mass index (BMI) were summarized using mean and standard deviation if normally distributed and median and interquartile ranges (IQR) if data was not normally distributed. Normality of distribution was assessed using Shapiro-Wilk test. These data were compared using Student’s *t* test if normally distributed, and Wilcoxon rank sum test if not normally distributed. Categorical variables were compared using the Pearson Chi-square (χ^2^) test and summarized as percentages (%). Multiple imputation with ten imputed datasets was used to deal with missing data. MICE is best practice when dealing with missing data and can provide unbiased estimates even with high levels of missingness, and some protection when data are missing not at random.^10^

Multivariable cox-models were used on ten imputed datasets to generate adjusted hazard ratios (aHR) with 95% confidence intervals (95% CI) for mortality over our study period. Our model was adjusted for the following characteristics: age, sex, ethnicity, year of admission, hospital region, heart rate, blood pressure, co-morbid conditions (hypertension, diabetes mellitus, history of asthma or COPD, history of cerebrovascular accident (CVA) (composite of transient ischaemic episodes, ischaemic, and haemorrhagic strokes), peripheral vascular disease (PVD) (The presence of PVD, either presently symptomatic or previously treated by intervention or surgery, including known renovascular disease and aortic aneurysm), hypercholesterolaemia, family history of coronary artery disease (CAD), smoking history, chronic renal failure, previous AMI, AF or flutter, current cancer, angina, previous PCI and previous CABG, inpatient revascularization by PCI or CABG), cardiac arrest, LV systolic function and Killip classification.

Hazard ratios shown are from comparison of patients with cardiac devices to those without. Separate Cox-models were run to create hazard ratios for 30-days, 1-year, 5-years and overall mortality. Kaplan-Meier curves were plotted to demonstrate unadjusted survival, and the Stcurve function was used in Stata 18.0 to illustrate adjusted survival, using the previously specified cox-model. Kaplan-Meier curves according to OBQI score are shown for patients that survived over 30 days from admission only. Cox-models were judged to be appropriate after assessment of Kaplan-Meier survival curves and confirmed by the assessment of the Schoenfeld residuals, demonstrating proportional hazard over our study period. Additionally, we calculated our landmark survival analysis from applying the previously specified survival models to the population of patients that survived beyond 30 days from index admission with STEMI, removing patients that died within 30 days of admission

A supplementary analysis was undertaken to investigate the odds of receiving reperfusion within 60 and 90 min of arrival to hospital, and within 120 min from ‘call for help’, which is defined as patient call to emergency services, depending on the presence of a cardiac device, adjusting for the previously specified covariates (excluding invasive coronary angiography and inpatient revascularization). We included only patients where all relevant data to calculate reperfusion timings was available for this supplementary analysis. A multivariate logistic regression model was applied to ten imputed datasets, to generate odds ratios with 95% Confidence Intervals (CIs). We conducted a further subgroup analysis to evaluate the impact of a ‘paced rhythm’, rather than just the presence of a cardiac device. We excluded all patients with missing QRS data from this section of the analysis (150 611). We formed our ‘likely paced rhythm’ group as being those with a broad QRS and a cardiac device at the time of admission, comparing their outcomes to patients with a cardiac device and a narrow QRS, described a ‘not paced at time of admission’, and all other patients without a cardiac device. All statistical analyses performed using the Stata version 18.0. Graphical abstract was created using a personal ‘Biorender’ license.

## Results

### Baseline characteristics

**Figure 1:**
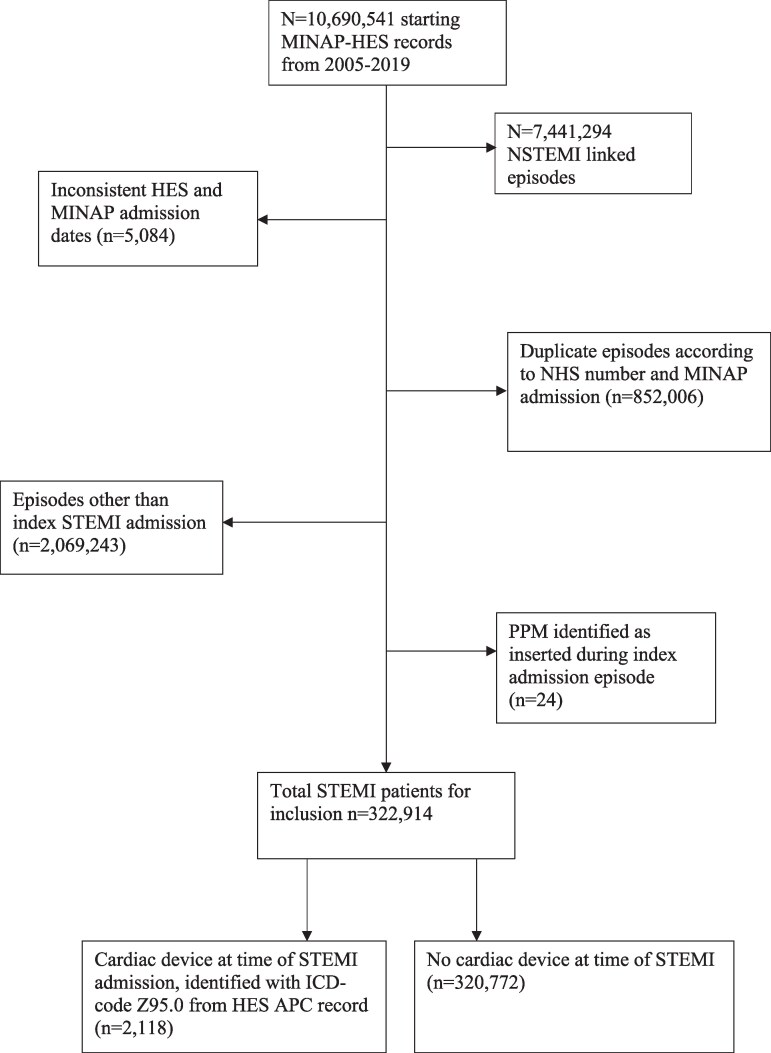
STROBE Diagram detailing study inclusion and exclusion criteria.

After applying our exclusion criteria, there were 322 890 patients with STEMI between January 2005 and March 2019. 2118 (0.7%) patients had a cardiac device at the time of admission. (*Figure 1*). Median follow-up time for included patients was 2338 days (1164d-3755d). Patients with a cardiac device were older [median age 78 (IQR 70–85) vs. 66 (IQR 56–77)], more likely to be female (32% vs. 29%, *P* < 0.001) and of White ethnicity (93% vs. 90%, *P* = 0.032) (*Table 1*). Patients with a cardiac device were more comorbid with conditions such as congestive cardiac failure (CCF) (15% vs. 2%), chronic renal failure (10% vs. 3%), and diabetes mellitus (23% vs. 16%) (all *P* < 0.001). Furthermore, patients with a cardiac device were more likely to have had previous AMI (35% vs. 12%), previous PCI (17% vs. 6%) and previous CABG (12% vs. 3%) (all *P* < 0.001).

**Table 1:** Demographic comparison between STEMI patients with or without cardiac device at time of presentation

Variable	Cardiac device at time of admission (*n* = 2118)	No cardiac device (*n* = 320 772)	*P*-value	Proportion missing (*n*%)
Age, years, median (IQR)	78 (70–85)	66 (56–77)	<0.001	0
Female (%)	673/2118 (32)	93 930/320 782 (29)	0.012	0
BMI, median [IQR]	25.9 (23.2–29.3)	26.8 (24.0–30.1)	<0.001	53
Ethnicity–White	1190/1285 (93)	145 882/161 795 (90)	0.032	49
Ethnicity—Asian	59/1285 (5)	9963/161 795 (6)		
Ethnicity—Black	7/1285 (1)	1374/161 795 (1)		
Ethnicity—Mixed and Other	29/1285 (2)	4576/161 795 (3)		
Killip Class				
No Heart Failure (%)	804/1135 (71)	115 975/140 646 (82)	<0.001	56
Basal crepitations (%)	187/1135 (16)	13 100/140 646 (9)		
Pulmonary oedema (%)	83/1135 (7)	5756/140 646 (4)		
Cardiogenic shock (%)	61/1135 (5)	5815/140 646 (4)		
Smoking				
Previous smoker (%)	681/1837 (37)	80 394/290 277 (28)	<0.001	10
Current smoker (%)	301/1837 (16)	107 257/290 277 (37)		
CCF (%)	275/1877 (15)	6858/279 739 (2)	<0.001	13
Hypercholesterolemia (%)	630/1835 (34)	81 572/278 370 (29)	<0.001	13
Diabetes mellitus (%)	462/1994 (23)	47 827/302 043 (16)	<0.001	6
Cerebrovascular disease (%)	266/1876 (14)	15 846/279 647 (6)	<0.001	13
History of angina (%)	586/1865 (31)	37 518/282 164 (13)	<0.001	12
Peripheral vascular disease (%)	107/1849 (6)	8475/277 821 (3)	<0.001	13
Chronic renal failure (%)	177/1855 (10)	8060/278 663 (3)	<0.001	13
Hypertension (%)	1054/1892 (56)	123 468/285 327 (43)	<0.001	11
Asthma/COPD (%)	265/1871 (14)	33 688/278 547 (12)	0.006	13
Family history of CAD (%)	331/1480 (22)	79 347/238 649 (33)	<0.001	26
Previous AMI (%)	649/1908 (34)	33 928/285 398 (12)	<0.001	11
Previous PCI (%)	327/1880 (17)	16 867/281 206 (6)	<0.001	12
Previous CABG (%)	230/1890 (12)	7367/281 351 (3)	<0.001	12
Heart rate, bpm, median (IQR)	75 (64–89)	76 (64–90)	0.389	12
Systolic blood pressure, median (IQR)	130 (110–150)	132 (114–151)	0.002	11
LVSD				
Good LV function (%)	341/1061 (32)	72 268/155 709 (46)	<0.001	51
Moderate LVSD (%)	440/1061 (41)_	65 209/155 709 (42)		
Severe LVSD (%)	280/1061 (26)	18 232/155 709 (12)		
Cardiac arrest (%)	335/2032 (16)	37 711/308 339 (12)	<0.001	4
Infarct site				
Anterior (%)	445/1195 (37)	80 018/207 949 (38)	<0.001	29
Inferior (%)	485/1195 (41)	98 752/207 949 (47)		
Lateral (%)	56/1195 (5)	8455/207 949 (4)		
Posterior (%)	36/1195 (3)	6070/297 949 (3)		
Broad QRS (%)	304/1125 (25)	13 018/180 420 (7)	<0.001	38
Atrial fibrillation (%)	647/2118 (31)	32 482/320 772 (10)	<0.001	0
Active cancer (%)	77/2118 (4)	7557/320 722 (2)	<0.001	0

Chronic renal failure is recorded in MINAP as those with serum creatinine chronically elevated above 200 micromol/L. Atrial fibrillation includes paroxysmal, permanent, chronic AF, and atrial flutter.

BMI, body mass index; CABG, coronary artery bypass graft; CAD, coronary artery disease; CCF, congestive cardiac failure; COPD, chronic obstructive pulmonary disease; GRACE, global registry of acute coronary events; IQR, interquartile range; LVSD, left ventricular systolic dysfunction; MI, myocardial infarction.

### Management and clinical outcomes

Patients with a cardiac device were less likely to be treated with aspirin (93% vs. 97%) and ACE inhibitors/ARB (72% vs. 78%), these patients were also less likely to be managed invasively with coronary angiography (66% vs. 77%), or PCI (54% vs. 66%) (all *P* < 0.001) compared with patients without a cardiac device (*Table 2*). Patients with a cardiac device were more likely to suffer inpatient mortality (16% vs. 8%) and have worse survival at 1 year (30% vs. 13% mortality) and 5 years (55% vs. 25% mortality) (all *P* < 0.001). There was no significant difference between major bleeding and reinfarction rates. Patients with a cardiac device had comparable rates of anterior infarction (37% vs. 38%, *P* < 0.001). Patients with a cardiac device were significantly more likely to have a broad QRS at admission (25% vs. 7%, *P* < 0.001).

**Table 2:** Management strategy and outcome comparison between STEMI patients with or without cardiac device at time of presentation

Variables	Cardiac device at time of admission (*n* = 2118)	No cardiac device (*n* = 320 772)	*P*-value	Proportion missing (*n*%)
Low molecular weight heparin (%)	787/1723 (46)	115 915/254 221 (46)	0.947	21
Fondaparinux (%)	271/1584 (17)	25 617/212 156 (12)	<0.001	34
Warfarin (%)	180/1705 (11)	9077/249 749 (4)	<0.001	22
Glycoprotein 2b/3a inhibitor (%)	204/1734 (12)	42 281/256 195 (17)	<0.001	20
IV Nitrate (%)	360/1698 (21)	59 066/250 220 (24)	0.020	22
Furosemide (%)	667/1715 (39)	51 237/249 765 (21)	<0.001	22
MRA (%)	209/1407 (15)	16 596/177 205 (9)	<0.001	45
Aspirin (%)	1958/2096 (93)	308 405/318 164 (97)	<0.001	1
P2Y12 inhibitor (%)	1696/2029 (84)	258 843/303 340 (85)	0.027	5
Statins (%)	1643/2078 (79)	261 576/315 627 (83)	<0.001	2
ACE inhibitors/ARB (%)	1492/2074 (72)	246 476/314 628 (78)	<0.001	2
Beta-Blockers (%)	1629/2081 (78)	252 970/315 406 (80)	0.028	2
Coronary angiogram (%)	1327/2008 (66)	235 480/304 151 (77)	<0.001	5
Percutaneous coronary intervention (%)	1124/2093 (54)	209 860/315 821 (66)	<0.001	2
CABG surgery (%)	16/1501 (1)	3248/236 874 (1)	0.310	26
Revascularization (CABG surgery/PCI) (%)	1137/2093 (54)	212 424/315 821 (67)	<0.001	2
Inpatient mortality (%)	338/2118 (16)	25 527/320 772 (8)	<0.001	0
One year mortality (%)	30%	13%	<0.001	0
Five-year mortality (%)	55%	25%	<0.001	19
Ten-year mortality (%)	74%	40%	<0.001	55
Reinfarction (%)	36/1911 (2)	6318/289 022 (2)	0.368	10
Major bleeding (%)	28/2052 (1)	3308/306 394 (1)	0.214	4

MACE is defined as composite endpoint of in-hospital death and reinfarction. 1-year, 5-years and 10-years mortality estimates derived from the Kaplan-Meier technique.

ACE, angiotensin-converting-enzyme; ARB, angiotensin receptor blockers; CABG, coronary artery bypass graft; IV, intravenous; MACE, major adverse cardiovascular events; MRA, mineralocorticoid receptor antagonist; PCI, percutaneous coronary intervention.

### Quality of care indicators

**Figure 2:**
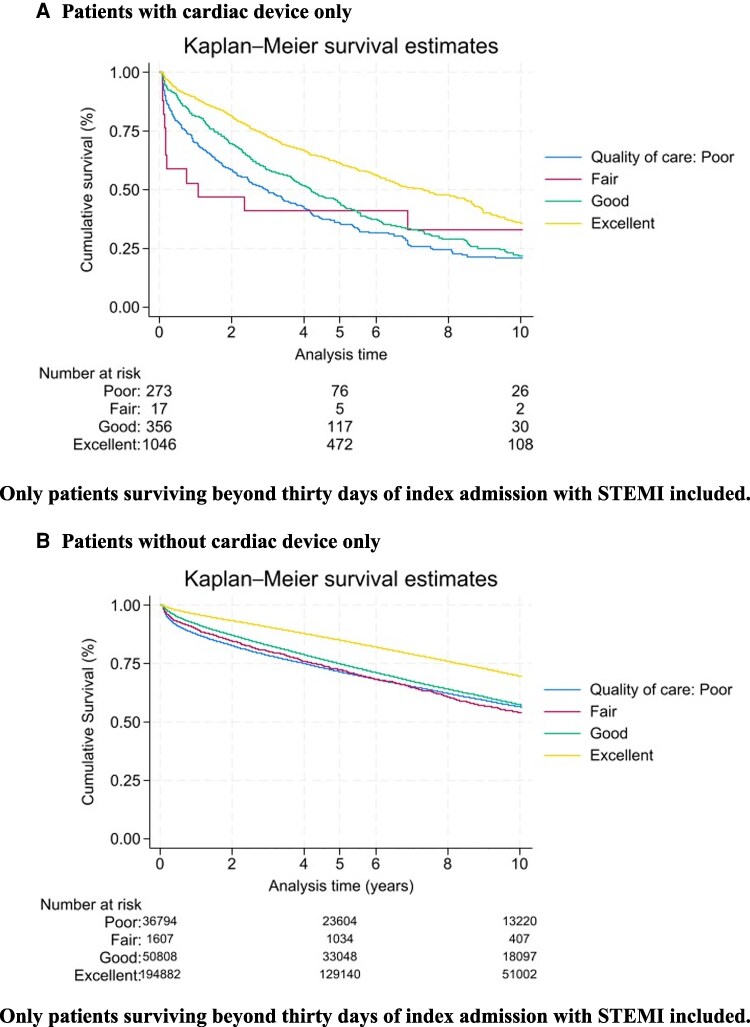
Kaplan-Meier Survival analysis for STEMI patients with and without cardiac devices according to inpatient quality of care. (*A*) Patients with cardiac device only. Only patients surviving beyond 30 days of index admission with STEMI included. (*B*) Patients without cardiac device only. Only patients surviving beyond 30 days of index admission with STEMI included.

Quality of care received by patients with cardiac devices was poorer, with 97% of these patients undergoing reperfusion within 12 h (vs. 99%, *P* < 0.001) and 76% with a ‘Door to balloon time’ of less than 90 min (vs. 86%, *P* < 0.001). A ‘Call to Balloon time’ of under 120 min was significantly less likely in patients with a cardiac device (42% vs. 60%, *P* < 0.001). Medical management also varied according to the presence of a cardiac device, patients with a cardiac device were less likely to receive dual antiplatelet therapy (DAPT) (80% vs. 83%, *P* < 0.001) and 79% receiving ACEi or ARB on discharge (vs. 84%, *P* < 0.001). The mean OBQI score for patients with cardiac devices was 81.7 vs. 86.1 for those without cardiac devices (*P* < 0.001) (*Table 3*). *Figure 2A* shows unadjusted mortality depending on inpatient quality of care received for patients with a cardiac device, and *Figure 2B* shows this for patients without cardiac device.

**Table 3:** Quality indicators for STEMI patients with or without cardiac device at time of presentation

Variables	Cardiac device at time of admission (*n* = 2118)	No cardiac device (*n* = 320 772)	*P*-value	Proportion missing (*n*%)
Reperfusion within 12 h (%)^a^	1067/1100 (97)	232 295/235 752 (99)	<0.001	27
‘Door to Balloon’ time <90 min (%)	834/1110 (76)	203 435/235 752 (86)	<0.001	27
‘Door to Balloon’ time <60 min (%)	631/1110 (57)	171 101/235 752 (73)	<0.001	27
Call to Balloon <120 min	422/1003 (42)	126 225/210 365 (60)	<0.001	35
DAPT received on discharge (%)	1635/2048 (80)	254 596/305 103 (83)	<0.001	5
ACEi or ARB on discharge for those with moderate and severe LVSD (%)	566/717 (79)	70 043/83 235 (84)	<0.001	0
OBQI^b^				
Mean OBQI score	81.7	86.1	<0.001	2
Cardiac rehabilitation (%)	1334/1873 (71)	241 537/285 710 (85)	<0.001	11

ACEi/ARB, angiotensin converting enzyme inhibitor/angiotensin receptor blockers; ACVC, Association for Acute Cardiovascular Care; CRUSADE, can rapid risk stratification of unstable angina patients suppress adverse outcomes with early implementation of the ACC/AHA guidelines; DAPT, dual antiplatelet therapy; EF, ejection fraction; ESC, European Society of Cardiology; GRACE, global registry of acute coronary events; LMWH, low molecular weight heparin; LV, left ventricle; LVSD, left ventricular systolic dysfunction; N/A, Not Available.

^a^Proportion of patients undergoing reperfusion within specified timeframes includes only patients with valid time of arrival to hospital and time of reperfusion, therefore the denominator is only inclusive of patients that have undergone reperfusion, not all included study patients.

^b^Opportunity based QI (The score consisted of 6 evidence-based processes of care: the prescription of aspirin, thienopyridine inhibitor, β-blocker, angiotensin converting enzyme inhibitor (ACEi), HMG CoA reductase enzyme inhibitor (statin) and enrolment onto a cardiac rehabilitation programme at the time of discharge). The OBCS reflects the number of care opportunities fulfilled at each hospital (numerator) divided by the number of opportunities to provide care (denominator). Excluded from both numerator and denominator were particular interventions that were contra-indicated, not applicable, not indicated in, or declined by, individual patients.

### Long-term mortality analysis

**Figure 3:**
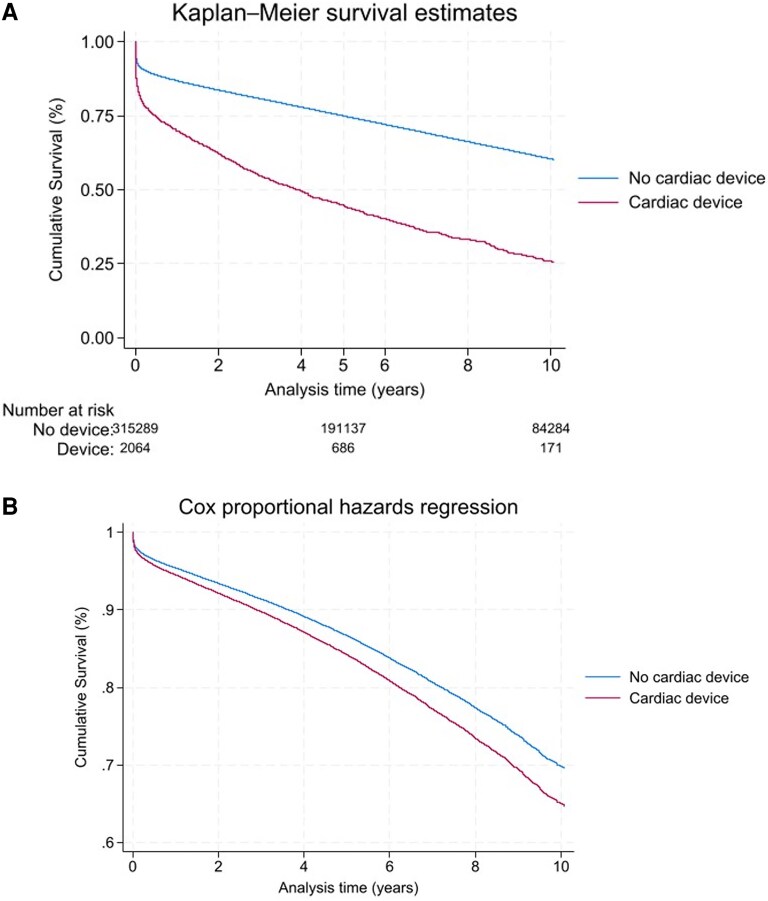
Kaplan-Meier and adjusted Survival analysis for STEMI patients with and without cardiac devices. *Cardiac devices refer to permanent pacemakers, CRT devices and implantable cardiac defibrillators, temporary venous pacing systems inserted during index admission are not included.

**Figure 4:**
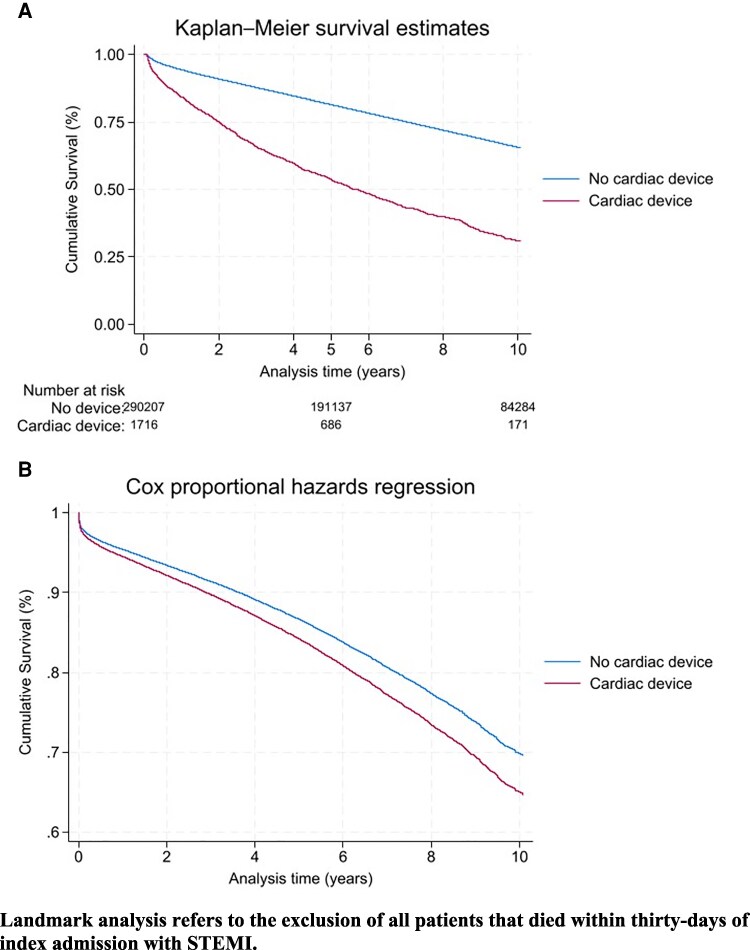
Landmark Kaplan-Meier and adjusted Survival analysis for STEMI patients with and without cardiac devices. Landmark analysis refers to the exclusion of all patients that died within 30-days of index admission with STEMI.

Patients with a cardiac device had a significantly higher adjusted risk of death at 5 years (aHR 1.12 95% CI 1.05–1.20, *P* < 0.001) and overall mortality (aHR 1.12 95% CI 1.06–1.10, *P* < 0.001), compared with those without cardiac devices (*Table 4*). Excluding all mortality within 30-days of admission with STEMI, patients with cardiac devices have a significantly higher adjusted risk of death at 1-year (aHR 1.19 95% CI 1.05–1.35, *P* = 0.007), 5-years (aHR 1.23 95% CI 1.13–1.34, *P* < 0.001) and overall (aHR 1.20 95% CI 1.12–1.29, *P* < 0.001) (*Table 5*). Long-term unadjusted mortality is displayed in Kaplan-Meier chart format in *Figure 3A*, and adjusted long-term mortality is illustrated over the same time-period in *Figure 3B*. *Figure 4A* shows unadjusted long-term survival for patients that survived over 30-days from admission with STEMI, and *Figure 4B* shows adjusted mortality over the same time-period, excluding any patients dying within 30-days of STEMI admission.

**Table 4:** Survival analysis comparison between STEMI patients with or without cardiac device at time of presentation

Outcome variables	Adjusted hazard ratio for patients with cardiac device compared those without (95% CIs)	*P*-value
Primary Outcomes		
Thirty-day mortality	1.01 (0.91–1.13)	0.835
One-year mortality	1.07 (0.98–1.16)	0.139
Five-year mortality	1.12 (1.05–1.20)	<0.001
Overall mortality	1.12 (1.06–1.19)	<0.001

Adjusted Hazard ratios are presented with 95% CIs, adjusted for: age, sex, ethnicity, year of admission, heart rate, blood pressure, co-morbid conditions (hypertension, diabetes mellitus, history of asthma or COPD, history of CVA or PVD, hypercholesterolaemia, family history of coronary artery disease, smoking history, chronic renal failure, previous AMI, atrial fibrillation or flutter, current cancer, angina, previous PCI and previous CABG, invasive coronary angiogram, inpatient revascularization by PCI or CABG), cardiac arrest, LV systolic function, Killip classification, and admission hospital region.

**Table 5:** Landmark survival analysis for STEMI patients with or without cardiac device at time of presentation

Outcome variables	Adjusted hazard ratio for patients with cardiac device compared those without (95% CIs)	*P*-value
Primary Outcomes		
One-year mortality	1.19 (1.05–1.35)	0.007
Five-year mortality	1.23 (1.13–1.33)	<0.001
Overall mortality	1.20 (1.12–1.29)	<0.001

Landmark refers to the exclusion of patients that died within 30 days of admission with STEMI. Adjusted Hazard ratios are presented with 95% CIs, adjusted for: age, sex, ethnicity, year of admission, heart rate, blood pressure, co-morbid conditions (hypertension, diabetes mellitus, history of asthma or COPD, history of CVA or PVD, hypercholesterolaemia, family history of coronary artery disease, smoking history, chronic renal failure, previous AMI, atrial fibrillation or flutter, current cancer, angina, previous PCI and previous CABG, invasive coronary angiogram, inpatient revascularization by PCI or CABG), cardiac arrest, LV systolic function, Killip classification, and admission hospital region.

### Supplementary analysis

**Figure 5:**
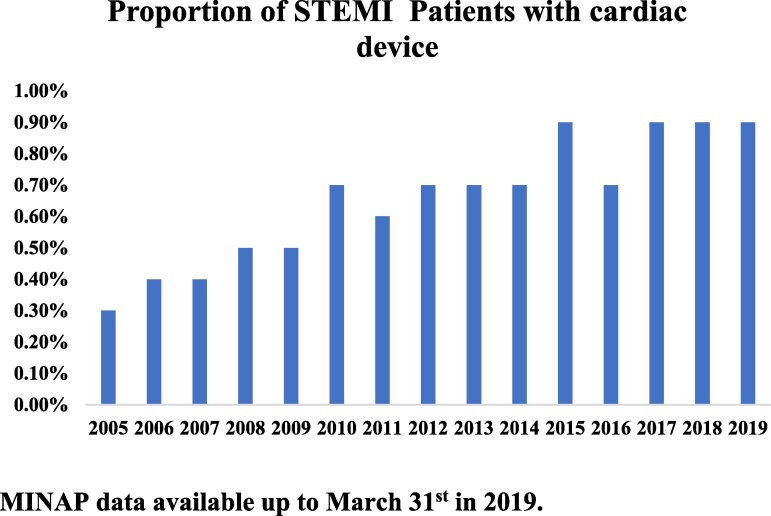
Total number of STEMI patients with cardiac device per year of study. MINAP data available up to March 31st in 2019.

*Figure 5* illustrates how the number of patients suffering STEMI with a cardiac device increased over our study period, from 0.3% in 2005 to 0.9% in 2018. Patients with cardiac devices were less likely to have a ‘door to balloon time’ of under 60 min (aOR 0.61 95% CI 0.54–0.70) and under 90 min (aOR 0.59 96% CI 0.51–0.69) (all *P* < 0.001) (see Supplementary material online, *Table S1*). The results of our supplementary analysis of patients presenting with a paced rhythm are presented in Supplementary material online, *Table S2*.

## Discussion

Our large-scale, contemporary national study investigating the quality of care and long-term mortality of patients with cardiac devices admitted with STEMI reports a range of important, clinically significant findings. Firstly, these patients differ demographically from the broader STEMI population, being significantly older and more likely to have prevalent cardiovascular comorbidities, such as hypertension, diabetes mellitus, or heart failure. This identifies them as a high-risk group for adverse outcomes following STEMI. Secondly, patients with a cardiac device received poorer quality care during their admission, being less likely to be treated with guideline-directed medical therapy such as with dual-antiplatelet therapy or ACE-inhibitors (GDMT) and were less likely to undergo PPCI for STEMI within specified timeframes, despite adjusting for important confounders such as age at admission, common cardiovascular comorbidities, and factors that may delay prompt reperfusion such as the presence of cardiogenic shock or cardiac arrest. Thirdly, the number of patients presenting with STEMI with a cardiac device increased during the study period, reflecting an increasingly older, multimorbid population.

Finally, these patients exhibited elevated risks of both short- and long-term mortality, even after adjusting for confounders. This risk was particularly pronounced when excluding early inpatient mortality (within 30 days), underscoring a persistent vulnerability beyond the acute phase.

There are limited studies investigating the outcomes of STEMI in patients with cardiac devices. The small number of previous studies in this area have important limitations in their generalizability to contemporary populations and are limited by a small sample size of patients with cardiac devices or lack data on long-term mortality outcomes.^6^ Ours is the largest contemporary study of STEMI patients with a cardiac device to date, giving an important insight into the care of this significant cohort of patients, that will continue to grow over the coming years

Our study demonstrates that patients with cardiac devices make up a small yet increasing proportion of STEMI admissions in England and Wales, with 0.7% of STEMI admissions over the study period having a cardiac device at the time of presentation. This progressively increased over the course of the study. This is in keeping with other contemporary studies which report a prevalence of cardiac devices of up to 1.0%, increasing in prevalence with recency of study.^7,8^ We suspect this is due to a combination of increasing prevalence of PPM implantation for bradyarrhythmia in an ageing population, but alongside increasing ICD and CRT implantation in the growing heart failure population.^5^ However, this population with cardiac devices is seldom mentioned in STEMI guidelines and clearly represent a challenge in management.

Our study has demonstrated that patients with cardiac devices are less likely to receive timely management of STEMI, demonstrated by lower rates of ‘Door to balloon’ time in < 60 min, 90 min, and ‘Call to balloon’ time of < 120 min, both in unadjusted data, but also after adjusting for a wide range of demographic features and comorbidity. We suggest that the reasons for this are complex, and multifactorial. Firstly, we suspect there are difficulties interpreting the initial ECG on presentation. It has previously been shown how STEMI may be challenging to diagnose in paced rhythms, given paced rhythms can interfere with Q-waves and cause marked repolarization abnormalities.^9^ Specific criteria such as the modified Sgarbossa criteria exist for diagnosing STEMI in patients with LBBB, which can as also be applied to RV paced rhythms, but the awareness and understanding of this is not always well established.^7^ Contemporary studies show up to 6 in 10 patients with cardiac devices presenting with AMI could be missed even when using scoring systems.^6^ Therefore, it is not uncommon for patients with cardiac devices presenting with STEMI to be initially diagnosed as NSTEMI due to diagnostic uncertainty with a paced rhythm.^8^ Additionally, the challenge of diagnosis of STEMI in a paced rhythm could lead to patients not being identified and pre-alerted as STEMI by emergency services, leading to assessments at non-primary PCI centres or in Emergency departments, rather than direct specialty cardiology assessment, which will lead to delays to reperfusion.^11^ We suggest that the combination of the slower identification of STEMI, misclassification of STEMI as NSTEMI and not being identified for appropriate primary PCI pathways could be contributors to the reduced odds of receiving guideline mandated PPCI for STEMI within the specified timeframes for patients with cardiac devices.

Our study demonstrates that STEMI patients with cardiac devices have poorer long-term outcomes (5 years and total study time to July 2021), compared with patients without cardiac devices, whereas, we do not see as strong an association with shorter-term outcomes (defined as 30 days and 1-year mortality). As with the delays to perfusion, the reasons for this will be multifactorial. We must acknowledge that although we are able to correct for a range of comorbidities in our statistical models, given the cardiac device cohort is significantly older, with higher proportions of most important comorbidities, there is a risk of residual confounding from variables that are outside of our models, and that the mortality differences observed are more reflective of the older, comorbid patient cohort. However, given our models are robust, with a significant number of variables included, we must consider additional reasons for the poorer survival in this cohort. Firstly, the reduced receipt of GDMT such as dual-antiplatelet therapy, ACE-inhibitors or ARBs and referral to a recognized cardiac rehabilitation program could be an important contributor, as we know that these factors are associated with poorer long-term outcomes post-STEMI.^12–15^ Additionally, we know that prompt reperfusion in STEMI is associated with improved long-term survival in STEMI, with reduced impairment of left ventricular (LV) dysfunction and reduced rates of mechanical complications, and delays to reperfusion in the device-therapy cohort could contribute to their poorer long-term survival.^3,4,16^ We must however acknowledge due to the elevated rates of complex multimorbidity, this cohort of patients with cardiac devices is a frail population with poorer left ventricular function, and higher-bleeding risk for example, and as such, patients may be unsuitable for an immediate invasive strategy due to frailty, and concerns regarding futility,^17–19^ and there may be reasonable bleeding concerns with DAPT or concerns with renal function for ACE inhibitors and ARBs for example. Overall, our finding of additional long-term mortality risk needs to be interpreted with caution given the demographic differences between the groups, and causality cannot be attributed to delays in reperfusion given our lack of data on procedural results and outcomes.

Further research should be targeted at improving the identification of STEMI in patients with a paced rhythm, addressing the disparity in access to high-quality STEMI care that we have demonstrated. Potential measures include transiently inhibiting pacing function to determine underlying rhythm, however this comes with risk in pacing dependent patients.^8^ Additionally, early bedside echocardiography in paced patients should be utilized to assess for regional wall abnormalities, which should be correlated with a history of cardiac chest pain in patients with a paced rhythm.^20^ There is additionally an important role in patient education for patients with cardiac devices, that although their device may be protective from their bradyarrhythmia for example, they remain at significant risk from myocardial infarction.

### Strengths

This study has several strengths. Firstly, the MINAP registry captures data from every STEMI admission across England and Wales. National analyses are more representative, meaning results can be generalizable. Secondly, this study provides the largest contemporary cohort of patients with cardiac devices presenting with STEMI, via our linkage to the HES Thirdly, given UK healthcare follows a publicly funded, universal healthcare model, disparities in care commonly found in insurance-based healthcare systems are minimized. Finally, the granular nature of the MINAP registry means many relevant confounders were adjusted for, including comorbidities and pharmacological management not commonly found in other datasets.

### Limitations

This study includes several limitations. Firstly, adverse events recorded on MINAP are self-reported with no external validation. Secondly, despite the broad number of variables included in MINAP, important confounders that could have mediated outcomes such as frailty, and CAD severity are not captured, and are therefore unable to be included in our models. The results of our study must therefore be interpreted in the context of potential residual confounding from additional comorbidities not included in our models or not recorded. Moreover, comorbidities recorded in MINAP are specific, meaning patients may or may not be coded for some conditions due to the definitions used within MINAP. Thirdly, whilst MINAP should capture data on type 1 AMI only, data is subject to misclassification, therefore a small number of patients with type 2 MI could be included, although this is far less likely for STEMI than NSTEMI. Although whether patients are taking or initiated on warfarin during their admission is recorded in MINAP, oral anticoagulation by other methods was not separately recorded, therefore with the increasing usage of direct oral anticoagulants (DOAC) over the study period, rates of antiplatelet therapy prescription should be interpreted with caution. Missing data is a common problem in large registry-based studies, although we did not include patients with missing data in key outcome variables in our study, the denominators for many of the variables in our descriptive Tables will be slightly different due to missing data. We elected to include patients from 2005 to 2019 in our study to maximize the number of patients with cardiac devices that we could include in our analysis, however we acknowledge that STEMI management has evolved over the study period, as have the indication for and types of device implanted, and that results for patients included earlier in the study may differ from those for patients included later in the study, which we display in Supplementary material online, *Table S3*.

Finally, patients with cardiac devices are a heterogenous group, and specific data on whether patients had a paced rhythm at the time of presentation is not available, and although we attempted to mitigate this using QRS duration at admission for a subgroup analysis, the included patient numbers were small. Additionally, device type or device indication data was not available, as we were unable to discern this from the linked HES and relevant ICD-10 codes, and we would expect that outcomes would vary according to device indication.

## Conclusion

The proportion of patients suffering STEMI with cardiac devices has increased over our study period. Patients with cardiac devices admitted with STEMI were more likely to be older and had more common cardiovascular comorbidities. Cardiac device patients were less likely to be managed with guideline directed medical therapy and were less likely to receive reperfusion therapy by primary PPCI within guideline mandated timeframes. Overall, patients with a cardiac device have poorer long-term survival post-STEMI over an extended follow-up period.

## Lead author biography


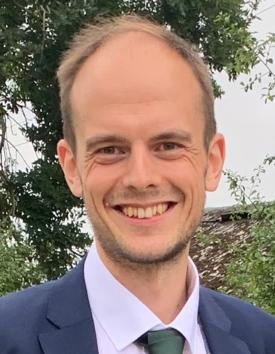


Nicholas Weight is a Clinical Research Fellow in Cardiology at Keele University, currently undertaking his PhD with the Keele Cardiovascular Research Group under the supervision of Professor Mamas Mamas. He completed his initial cardiology training at the University Hospital North Midlands as part of the integrated clinical academic training programme (NIHR ACF) and will return to subspecialty training in interventional cardiology after completion of his research period.

## Supplementary Material

oeaf139_Supplementary_Data

## Data Availability

The authors do not have authorization to share the data, but it can be accessed through contacting the National Institute for Cardiovascular Outcomes Research (NICOR) upon approval.
