# Supplementary material for: The quality of care and long-term mortality of patients with ST-elevation myocardial infarction and cardiac devices: a nationwide cohort study
Source: Eur Heart J Open. 2025 Oct 23;5(6):oeaf139. doi: 10.1093/ehjopen/oeaf139 (PMC12596147; doi:10.1093/ehjopen/oeaf139)
Supplement: oeaf139_Supplementary_Data [file oeaf139_supplementary_data.docx]

**Supplementary Table 1: Odds of undergoing revascularisation within guideline mandated time frames according to presence of cardiac device at time of STEMI**

| **Outcome variables** | **Odds ratio for undergoing revascularisation within specified time frame based on presence of cardiac device, compared to those without device (95% CIs)** | **P-value** |
| --- | --- | --- |
| **Primary Outcomes** | | |
| **Door to Balloon time <60 minutes** | 0.61 (0.54-0.70) | <0.001 |
| **Door to Balloon time <90 minutes** | 0.59 (0.51-0.69) | <0.001 |
| **Call to balloon time <120 minutes** | 0.68 (0.59-0.77) | <0.002 |

Adjusted odds ratios are presented with 95% CIs, adjusted for: age, sex, ethnicity, year of admission, heart rate, blood pressure, co-morbid conditions (hypertension, diabetes mellitus, history of asthma or COPD, history of CVA or PVD, hypercholesterolaemia, family history of coronary artery disease, smoking history, chronic renal failure, previous AMI, angina, previous PCI and previous CABG, cardiac arrest, LV systolic function, Killip classification, and admission hospital.

**Supplementary Table 2:** **Survival analysis comparison between STEMI patients with or without cardiac device at time of presentation**

| **Outcome variables** | **Adjusted hazard ratio for patients with cardiac device, stratified by QRS duration, compared those without (95% CIs)** | | | |
| --- | --- | --- | --- | --- |
| **Primary outcomes** | **Likely-paced rhythm (n=406)** | **P-value** | **Not-paced (n=896)** | **P-value** |
| **Thirty-day mortality** | 1.20 (0.95-1.50) | 0.122 | 1.22 (1.01-1.46) | 0.034 |
| **One-year mortality** | 1.10 (0.92-1.32) | 0.281 | 1.16 (1.01-1.33) | 0.040 |
| **Five-year mortality** | 1.15 (1.00-1.32) | 0.050 | 1.19 (1.07-1.32) | 0.002 |
| **Overall mortality** | 1.11 (0.98-1.26) | 0.113 | 1.17 (1.06-1.30) | 0.002 |

Adjusted Hazard ratios are presented with 95% CIs, adjusted for: age, sex, ethnicity, year of admission, heart rate, blood pressure, co-morbid conditions (hypertension, diabetes mellitus, history of asthma or COPD, history of CVA or PVD, hypercholesterolaemia, family history of coronary artery disease, smoking history, chronic renal failure, previous AMI, angina, previous PCI and previous CABG, invasive coronary angiogram, inpatient revascularisation by PCI or CABG), cardiac arrest, LV systolic function, Killip classification, and admission hospital.

“Likely-paced” refers to patients with a cardiac device at the time of admission, where a broad QRS is recorded on admission. Not-paced refers to patients with a cardiac device at the time of admission, with a narrow QRS recorded on admission. Patients where QRS duration is either recorded as unknown or missing are not included in this section of the analysis.

**Supplementary Table 3: Survival analysis comparison between STEMI patients with or without cardiac device at time of presentation for study periods (early, middle and late)**

| **Outcome variables** | **Adjusted hazard ratio for patients with cardiac device (95% CIs)** | **P-value** | **Adjusted hazard ratio for patients with cardiac device (95% CIs)** | **P-value** | **Adjusted hazard ratio for patients with cardiac device (95% CIs)** | **P-value** |
| --- | --- | --- | --- | --- | --- | --- |
|  | **Early study (2005-2009)** | | **Middle study (2010-2014)** | | **Late study (2015-2019)** | |
| **Thirty-day mortality** | 0.87 (0.68-1.11) | 0.263 | 0.95 (0.79-1.15) | 0.591 | 1.20 (1.02-1.43) | 0.033 |
| **One-year mortality** | 1.00 (0.84-1.21) | 0.965 | 1.03 (0.90-1.19) | 0.627 | 1.16 (1.01-1.33) | 0.032 |
| **Overall mortality** | 1.07 (0.96-1.21) | 0.233 | 1.12 (1.02-1.23) | 0.017 | 1.19 (1.08-1.33) | 0.001 |

Adjusted Hazard ratios are presented with 95% CIs, adjusted for: age, sex, ethnicity, year of admission, heart rate, blood pressure, co-morbid conditions (hypertension, diabetes mellitus, history of asthma or COPD, history of CVA or PVD, hypercholesterolaemia, family history of coronary artery disease, smoking history, chronic renal failure, previous AMI, atrial fibrillation or flutter, current cancer, angina, previous PCI and previous CABG, invasive coronary angiogram, inpatient revascularisation by PCI or CABG), cardiac arrest, LV systolic function, Killip classification, and admission hospital.

**Comparison is to patients without cardiac device at time of presentation.**
